# Developmental patterning of glutamatergic synapses onto retinal ganglion cells

**DOI:** 10.1186/1749-8104-3-8

**Published:** 2008-03-26

**Authors:** Josh L Morgan, Timm Schubert, Rachel OL Wong

**Affiliations:** 1 Department of Biological Structure, University of Washington, Seattle, WA 98195, USA

## Abstract

**Background:**

Neurons receive excitatory synaptic inputs that are distributed across their dendritic arbors at densities and with spatial patterns that influence their output. How specific synaptic distributions are attained during development is not well understood. The distribution of glutamatergic inputs across the dendritic arbors of mammalian retinal ganglion cells (RGCs) has long been correlated to the spatial receptive field profiles of these neurons. Thus, determining how glutamatergic inputs are patterned onto RGC dendritic arbors during development could provide insight into the cellular mechanisms that shape their functional receptive fields.

**Results:**

We transfected developing and mature mouse RGCs with plasmids encoding fluorescent proteins that label their dendrites and glutamatergic postsynaptic sites. We found that as dendritic density (dendritic length per unit area of dendritic field) decreases with maturation, the density of synapses along the dendrites increases. These changes appear coordinated such that RGCs attain the mature average density of postsynaptic sites per unit area (areal density) by the time synaptic function emerges. Furthermore, stereotypic centro-peripheral gradients in the areal density of synapses across the arbor of RGCs are established at an early developmental stage.

**Conclusion:**

The spatial pattern of glutamatergic inputs onto RGCs arises early in synaptogenesis despite ensuing reorganization of dendritic structure. We raise the possibility that these early patterns of synaptic distributions may arise from constraints placed on the number of contacts presynaptic neurons are able to make with the RGCs.

## Background

Glutamatergic inputs provide the major excitatory drive onto neurons in the central nervous system (CNS). Much is known about how individual glutamatergic synapses are formed, maintained or eliminated during development [1-3]. The physiological function of postsynaptic cells, however, does not depend solely on how individual synapses are assembled. It also depends on the how the correct number, density, and often spatial distribution of these synapses are established across the dendritic arbor. How distinct spatial patterns of glutamatergic synaptic inputs are established during development is poorly understood. Addressing this fundamental issue appears more tractable in the retina than elsewhere in the CNS, largely because the functional input/output characteristics of its neurons have been well characterized and correlated with cellular morphology and circuitry [4].

In the vertebrate retina, many of the functional properties of retinal ganglion cells (RGCs) are determined by the lateral and vertical distribution of their inputs from bipolar cells (BCs). BCs that depolarize (ON) or hyperpolarize (OFF) in response to increased illumination stratify their axonal arbors at different depths within the inner plexiform layer (IPL; Additional file 1a). The laminar position of RGC dendrites within the IPL thus largely determines which functional type of BCs they contact. The axon terminal arbors of each subtype of BC also form non-overlapping mosaics in the IPL that maintain the spatial relationships of the inputs they receive from photoreceptors (Additional file 1b) [5-8]. Therefore, the lateral distribution of the RGC dendritic arbor and the inputs it receives from the overlying bipolar cell axonal mosaic largely determines the region of visual space sampled by the RGC (Additional file 1a,b). In primate [9] and rabbit [10,11] retina, the distribution of BC inputs across the dendritic arbor of large field RGCs is relatively uniform. In contrast, the distribution of BC inputs onto the dendritic arbor of large field alpha RGCs in cat retina peaks near the center of the dendritic field [12] (Additional file 1d). This synaptic gradient matches the spatial sensitivity profile of the alpha RGC's physiological receptive field center [12]. How the pattern of BC synaptic input is thought to give rise to the dome-shaped receptive field center of the large-field RGCs is shown in Additional file 1c–f.

How RGCs attain their mature spatial patterns of glutamatergic synaptic input, and thus their spatial receptive field profile, is not known. This is largely because, like other CNS neurons, it is difficult to reconstruct all the synapses onto an individual cell using traditional methods, such as serial electron microscopy. We adopted a different strategy to more rapidly obtain synaptic distribution maps for developing and mature RGCs. We biolistically transfected mouse RGCs with td-Tomato and PSD95 fluorescently tagged with yellow fluorescent protein (PSD95-YFP) in order to label their dendrites and glutamatergic postsynaptic densities, respectively. Transient expression of fluorescently tagged PSD95 has been used to visualize glutamatergic synapses in several systems [11,13-16]. In the retina, endogenous [17] and fluorescently tagged PSD95 [11,13] have been found postsynaptic to BC terminals. We examined the distribution of PSD95-YFP puncta on RGCs from P5, just before they are known to receive functional glutamatergic inputs [18], until the first postnatal month when the arbors are morphologically mature [19]. In order to count large numbers of synaptic puncta and to obtain spatial maps of synaptic input across the RGC dendritic arbors, we developed a semi-automated Matlab program. We took advantage of the fact that bistratified (ON and OFF) RGCs and monostratified large field RGCs have different spatial patterns of inputs to ascertain whether such differences are shaped largely by remodeling during development, or whether they are established from the onset of synaptogenesis.

## Results

**Figure 1:**
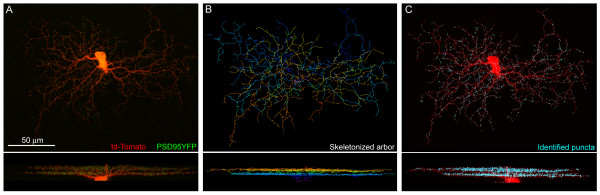
RGC dendrites and putative glutamatergic postsynaptic sites visualized upon expression of td-Tomato and PSD95-YFP. **(a) **Whole-mount or *en face *view (top) and orthogonal projection (bottom) of a P7 bistratified RGC transfected with td-Tomato and PSD95-YFP. **(b) **Skeletonization of the cell, color-coded for depth. The ON sublamina is blue; OFF sublamina is orange. **(c) **Identification of PSD95-YFP puncta (cyan dots) by a semi-automated dotfinder program (see Figure 2). Td-Tomato dendritic fill is shown in red.

**Figure 2:**
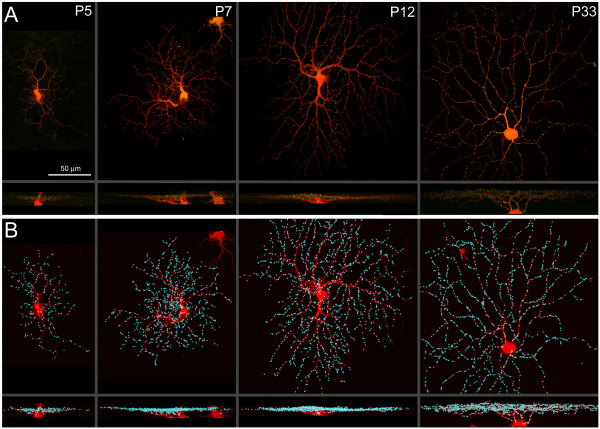
PSD-96-YFP puncta are present throughout the RGC dendritic arbor across synaptogenesis. **(a) ***En face *view (top) and orthogonal projections (bottom) of monostratified RGCs at various ages, expressing td-Tomato and PSD95-YFP. **(b) **Cells in (a) with puncta identified by the dotfinder program are shown in cyan.

Biolistic transfection of RGCs with td-Tomato and PSD95-YFP resulted in dendritic filling and punctate labeling of presumed glutamatergic postsynaptic sites after 18–24 hours, at all ages studied (Figures 1 and 2; Additional file 2). Using custom Matlab software we were able to map the distribution of dendritic length and putative synapses across dendritic arbors in three dimensions (Figures 1 and 2; and see Materials and methods). We focused our analysis on RGCs with relatively large cell bodies and dendritic arbors because they were more readily labeled by biolistic transfection. These RGCs (n = 71) were classified as monostratified or bistratified. Monostratified cells were further classified as ON (inner 3/5 of IPL) or OFF (outer 2/5 of IPL) according to the depth of their dendritic stratification. At P5, we did not observe any RGCs with clearly separated ON and OFF arbors (bistratified cells). Thus, for this early age, we pooled cells into a single group.

### PSD95-YFP as a marker of postsynaptic sites

**Figure 3:**
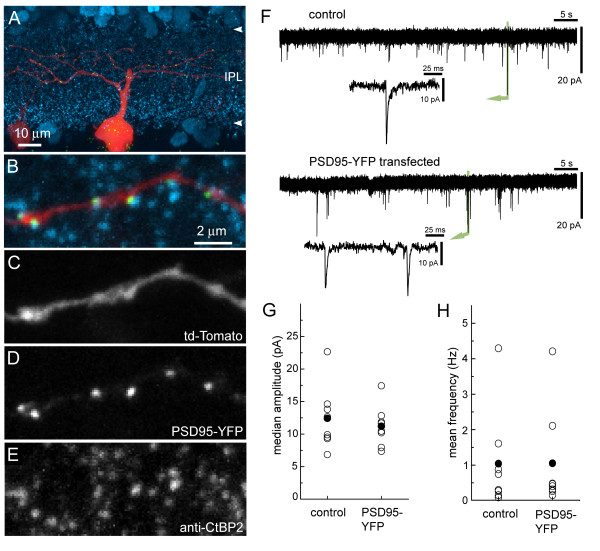
PSD95-YFP clustering appears to reflect normal distribution of postsynaptic densities. **(a) **Retinal cross-section showing a P35 RGC transfected with td-Tomato and PSD95-YFP. The section was immunostained for CtBP2 (cyan). **(b-e) **Higher magnification of a dendritic branch from the cell in (a) showing colocalization of PSD95-YFP puncta (b, d; green) with CtBP2 puncta (b, e; blue). **(f) **Example of whole-cell recordings showing sEPSCs in untransfected (control) and transfected RGCs at P12. **(g) **Median amplitude and **(h) **mean frequency of each control and transfected RGC (open circles). The mean of each group is shown by a black circle. N = 8 cells for each group

Immunolabeling of glutamatergic inputs onto RGCs was used to determine whether PSD95-YFP puncta faithfully labeled postsynaptic sites. The ribbons of BC presynaptic terminals were labeled with an antibody directed against carboxy-terminal binding protein 2 (CtBP2) [20] in P21 retinal cross sections (Figure 3a–e). Eleven cells with a total of 1,300 puncta were evaluated for colocalization of pre- and postsynaptic labels. Significant colocalization was quantified by comparing the observed distribution of PSD95-YFP puncta with distributions of puncta randomly positioned across the dendritic arbor (see Materials and methods). Because of the high density of presynaptic labeling in retinal cross sections, the colocalization criterion was arbitrarily defined as the lowest threshold that would still produce <50% colocalization in the randomized distribution of PSD95-YFP puncta. This method of defining colocalization produced a mean colocalization of 46 ± 1% in randomized puncta distributions and 78 ± 4% in the observed puncta. Ten of the eleven cells tested in this way had *P*-values < 0.001. The significant colocalization of PSD95-YFP puncta with presynaptic ribbons argued that PSD95-YFP could be used as a reasonable marker of glutamatergic postsynaptic sites on RGC dendrites.

In many cells, puncta were observed outside the main dendritic lamination (Additional files 3 and 4). In some adult OFF RGCs we observed PSD95-YFP puncta on primary dendrites within the ON sublamina (Additional file 3). We also observed such puncta colocalized with CtBP2 labeling (data not shown). Interestingly, bipolar inputs onto the primary processes outside of laminated arbor of RGCs in cat are absent or rare [21-23].

We also examined colocalization of PSD95-YFP puncta with VGlut1, the vesicular glutamate transporter found in BC terminals prior to the formation of ribbon synapses [18]. We found PSD95-YFP puncta that were apposed to immunolabeled VGlut1 positive terminals at P7 (data not shown), although the small number of BC axons labeled at this age made it difficult to quantify colocalization.

### PSD95-YFP expression does not alter spontaneous excitatory postsynaptic current characteristics of RGCs

To determine whether PSD95-YFP expression in RGCs significantly altered synaptic physiology, whole-cell patch clamp recordings were obtained from P12 RGCs transfected with PSD95-YFP/tdTomato plasmid (n = 8), and from neighboring untransfected (n = 8) cells in retinal wholemounts (Figure 3f). The average spontaneous excitatory postsynaptic current (sEPSC) amplitude of the transfected cells was 11.2 ± 1.1 pA, similar to that of the control cells at 12.4 ± 1.7 pA (*P *= 0.958; Figure 3g). The mean frequencies of sEPSCs of control (1.04 ± 0.50 Hz) and transfected (1.05 ± 0.50 Hz) RGCs were also not significantly different (*P *= 0.875; Figure 3h). These findings suggest that the PSD95-YFP expression in the transfected RGCs did not produce significant physiological changes and, in light of our immunolabeling results, indicate that the distributions of PSD95-YFP puncta observed here reflect well the endogenous patterns of glutamatergic postsynaptic sites on RGC dendrites.

### Growth and stratification of RGC dendritic arbors during synaptogenesis

**Figure 4:**
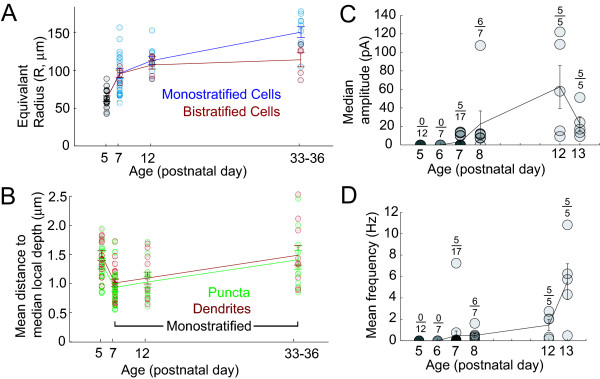
Growth and functional development of RGCs. **(a) **Increase in the dendritic arbor radius for monostratified and bistratified RGCs with age. **(b) **Stratification of puncta and dendrites of P5 and monostratified cells (P7–P33). **(c) **Median amplitude of sEPSCs recorded across ages. The number of cells that exhibited sEPSCs is noted above the total number of cells recorded at each age. (d) Mean frequency of sEPSCs recorded across ages. Error bars indicate standard error.

Before we determined how glutamatergic postsynaptic sites become organized into their mature patterns in RGCs, we first examined the growth and stratification of RGC dendritic arbors across development. To compare the lateral growth of dendritic arbors of the population of RGCs with maturation, we generated a disk that encompassed 98% of the total dendritic length and whose center was at the cell body. The radius of this circle (equivalent radius) was then plotted for monostratified and bistratified cells across ages (Figure 4a). Dendritic arbors of the RGCs were found to rapidly expand between P5 and P7, and continued to grow at a slower rate until adulthood. This developmental time-course matches that found in a previous study [19].

We next determined the distribution patterns of RGC dendrites and their synaptic puncta according to depth within the IPL. In order to reduce the influence of irregularities in the tissue, we obtained a stratification index as follows: for every 0.5 μm segment of the skeletonized arbor (each arbor comprised about 1,800–14,000 segments), we centered a circle of 30 μm radius on that segment and obtained the median depth of all segments within this sampling area. We then calculated the absolute distance of each dendritic segment from this median. The cell's stratification index was then the mean of all of these distances (shown for all cells in Figure 4b). The region of the dendritic arbor within 10 μm of the cell body was excluded from this analysis to remove primary dendrites. It was only between P5 and P7 that a significant narrowing in dendritic stratification was evident (P5: mean stratification index (μm) = 1.53 ± 09, n = 14 cells; P7: mean stratification index = 1.05 ± 0.07, n = 20 cells; *P *= 0.001; Figure 4b). Because, at P5, it was not possible to distinguish between monostratified and bistratified cells, it is difficult to determine the extent to which this change is due to a narrowing of monostratified arbors. After P7, the arbors of monostratified cells did not become more narrowly stratified, but rather appeared to expand somewhat (P33–36: mean stratification index (μm) = 1.54 ± 0.12, n = 10; *P *= 0.015). This pattern of stratification is consistent with that reported by Coombs *et al *[19]. It is important to note that while the width of the IPL occupied by the dendritic arbor does not decrease after P7, the percentage of IPL depth occupied by the dendrites of each cell decreases slightly because the total depth of the IPL increases well into the third postnatal week [19].

In order to determine whether contacts are formed within a narrower depth compared to the dendrites, we performed the same measure of stratification on the puncta as described for dendrites, except that each punctum was referenced to the median depth of nearby puncta. Such stratified contacts might be expected if glutamatergic synapses were directing RGC stratification. We found that the stratification index of puncta was, on average, 92 ± 2% as wide as the stratification index of dendrites. Relative to the ratio between punctum and dendritic stratification we observed in the adult (95 ± 2%), we did not find that the ratio was significantly greater at P5 (90 ± 2%, *P *= 0.253) and P7 (91 ± 2%, *P *= 0.322). Thus, from the earliest ages examined, PSD95-YFP puncta were evenly distributed across the depth of the stratified dendritic arbor.

### Recombinant PSD-95 is clustered and distributed across the RGC arbor prior to emergence of robust spontaneous glutamatergic neurotransmission

We found that PSD95-YFP formed distinct clusters within RGC dendritic arbors by P5, suggesting that glutamatergic synapses may be present earlier than previously expected [18]. We therefore recorded spontaneous postsynaptic currents from RGCs in a slice-preparation from P5 to P13 by voltage-clamping the cells at -60 mV. In these slice recordings cholinergic driven spontaneous bursting activity in RGCs was rarely observed.

Under our recording conditions, P5–6 RGCs did not demonstrate any sEPSCs (n = 19). In contrast, approximately half of these cells (52.6%, 10 of 19 cells) had distinct spontaneous inhibitory postsynaptic currents when held at 0 mV. These currents could be blocked with a combination of 2-(3-carboxypropyl)-3-amino-6-(4-methoxyphenyl) pyridazinium bromide (SR-95531; 5 μM), 1,2,5,6-tetrahydropyridin-4-yl methylphosphinic acid hydrate (TPMPA; 75 μM) and strychnine (0.5 μM) (n = 3). sEPSCs were first observed in a subset of P7 RGCs (Figure 4c,d). By P12, sEPSCs were robust and present in all cells. sEPSCs were abolished in the presence of the ionotropic glutamate receptor antagonists 6-cyano-7-nitroquinoxaline-2, 3-dione (CNQX) and DL-2-amino-7-phosphonoheptanoic acid (AP-7) at all ages (n = 17 cells, not shown).

In order to determine whether ionotropic glutamate receptors were expressed by RGCs at P5, prior to the emergence of sEPSCs, we recorded the responses of P5 RGCs to a puff of kainic acid (100 μM, 5 s). Cadmium chloride (250 μM) was present in the extracellular solution to eliminate synaptic transmission. Kainic acid evoked large inward currents (-116.18 ± 69.44 pA) in all five P5 cells. Application of CNQX (20 μM) and AP-7 (40 μM) during the kainate puff reduced the mean amplitude to -2.84 ± 2.69 pA (n = 5; *P *< 0.008). Thus, RGCs express functional glutamate receptors by P5 but spontaneous glutamatergic neurotransmission appears to be minimal, if at all present, at this age.

### Developing RGCs maintain constant synaptic sampling of IPL area despite dendritic remodeling and synaptogenesis

**Figure 5:**
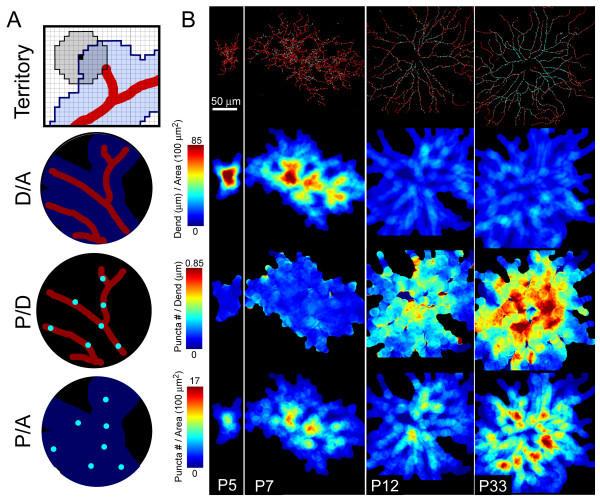
Spatial maps of dendritic and postsynaptic densities of RGCs across development. **(a) **Dendritic territories are determined by convolving a 10 μm diameter disk centered at every pixel of the image, with the dendritic skeleton (see Materials and methods). Within the dendritic territory of each cell, dendritic length (μm) per 100 μm^2 ^(D/A), puncta per 100 μm^2 ^(P/A), and puncta per micrometer of dendrite (P/D) were obtained. **(b) **Maps of local values of D/A, P/D, and P/A of OFF monostratified RGCs at different ages. Local densities were determined by counting puncta number and measuring dendritic (Dend) length within a sliding window with a diameter of 20 μm centered at each pixel of the image (see Materials and methods).

The dendritic arbors of RGCs can sample from one to hundreds of BC axonal terminals whose mosaic arrangement in the IPL provides a two-dimensional map of visual space [5,24]. We first define the RGC dendritic territory as a two-dimensional area (A) of the retina covered by its dendrites. This territory was obtained by convolving a two-dimensional projection of its dendritic skeleton with a 10 μm diameter disc (average size of a bipolar axon terminal), and then filling in holes in the field (see Materials and methods; Figure 5a, blue region).

We next quantified the sampling of visual space (Areal puncta density = Number of puncta/Dendritic territory (P/A)) for large monostratified RGCs across development. We then determined how these sampling fields relate to the distribution of dendrites (dendritic length/dendritic territory (D/A)) and to the density of postsynaptic densities on these dendrites (Linear puncta density = Number of puncta/Dendritic length (P/D)). Figure 5a illustrates how each of these measures was obtained. Figure 5b shows how synaptic puncta and dendritic densities are distributed across the dendritic field of representative monostratified RGCs across development.

**Figure 6:**
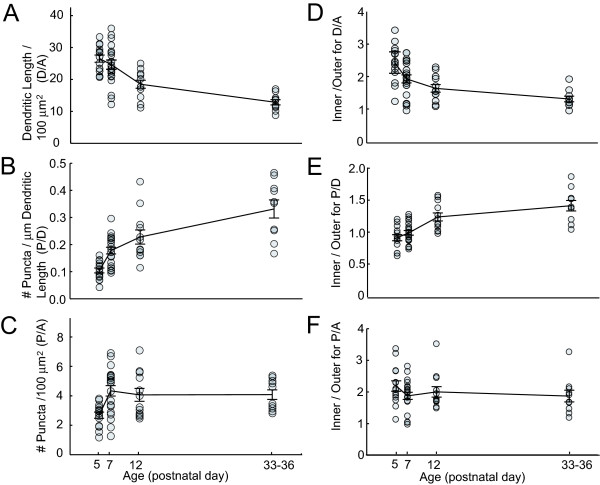
Dendritic and postsynaptic puncta densities across development. **(a-c) **Global dendritic and puncta densities for monostratified and P5 RGCs. Error bars = standard error of mean. **(d-f) **Ratio of dendritic and puncta densities of the inner half of the dendritic arbor divided by the outer half.

The average dendritic density of a RGC is then obtained by dividing the total dendritic length by the total area of the dendritic territory (Figure 6). RGCs with more densely branched dendritic arbors would have relatively higher dendritic densities. Likewise, average areal and linear synaptic densities were obtained by dividing the total number of puncta by the total area of the dendritic territory, or by the total dendritic length, respectively (Figure 6). Quantification of the population of monostratified cells (P7 to adult) demonstrates that there is an increase in the linear density of postsynaptic densities with age (ANOVA: *P *< 0.001; Figure 6b). However, this increase in linear puncta density did not appear to produce a corresponding increase in areal synaptic density after P7 (ANOVA: *P *= 0.449; Figure 6c). This relative stability in sampling density can be explained by the decrease in the dendritic density with age (ANOVA: *P *< 0.001; Figure 6a).

In addition to global changes in the density of dendrites and puncta, maps of dendritic and synaptic distributions often revealed centro-peripheral gradients in the developing RGCs (Figure 5b). We quantified these gradients by comparing dendritic and puncta densities for the inner and outer halves of a circle encompassing 98% of the dendritic arbor. In order to only compare distributions across the stratified arbor, the analysis excluded the region of the dendritic field within 10 μm of the cell body where the primary processes elaborated. We then plotted the ratio of the inner versus outer (central to peripheral) values as a function of age (Figure 6d–f). The dendritic gradient decreased from P7 to maturity (ANOVA: *P *= 0.004) whereas the gradient in linear puncta density increased with age (ANOVA: *P *< 0.001). The gradient in areal puncta density was highest at P5, but from P7 to adulthood it remained unchanged (ANOVA: *P *= 0.877). Thus, similar to global measures of puncta and dendrite densities, the mature centro-peripheral gradient in areal puncta density appears early in synaptogenesis, prior to the maturation of the gradient in dendritic density. The average dendritic and synaptic densities as well as centro-peripheral gradients across ages are plotted separately for ON and OFF monostratified RGCs in Additional file 5.

**Figure 7:**
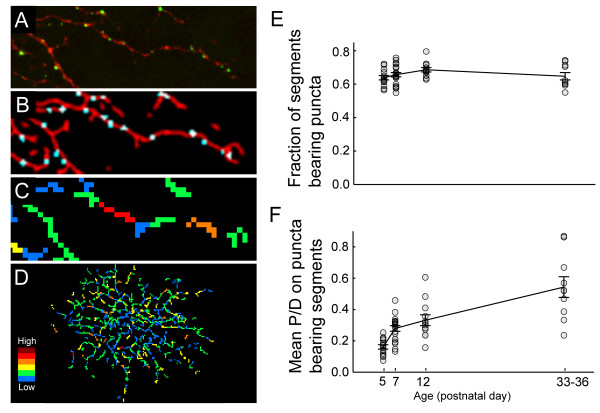
Local density of synaptic puncta across the RGC arbor. **(a-d) **The local density of synaptic puncta is determined by counting the number of puncta per dendritic segment. The length of the dendritic segment is determined for each cell by dividing the total length of the arbor by the total number of puncta: (a) raw image; (b) dendritic skeleton and puncta; (c, d) dendritic segments color coded according to synaptic density. Hot colors represent relatively higher puncta densities. **(e) **Plot of the proportion of segments bearing puncta for monostratified and P5 cells. Error bars = standard error of mean. **(f) **Plot of P/D for segments bearing puncta for monostratified and P5 cells.

In order to determine whether the increase in the linear density of puncta was due to the selective removal of branches without puncta, a more detailed analysis was performed on local synaptic densities. To this end, arbors were resampled in segment lengths equal to the total dendritic length divided by the total number of puncta (Figure 7a–d). We found that the proportion of segments on which puncta could be found remained constant throughout development (Figure 7e). When we plotted the linear density of puncta for those segments that possessed them, we observed an increase in density comparable to the increase observed for global measures of linear puncta density (Figure 7f). Together, these observations suggest that the global increase in the linear density of puncta is due to local increases in linear puncta density, not the selective elimination of processes without puncta.

### Development of postsynaptic glutamatergic sites on functionally distinct ON and OFF arbors of bistratified RGCs

**Figure 8:**
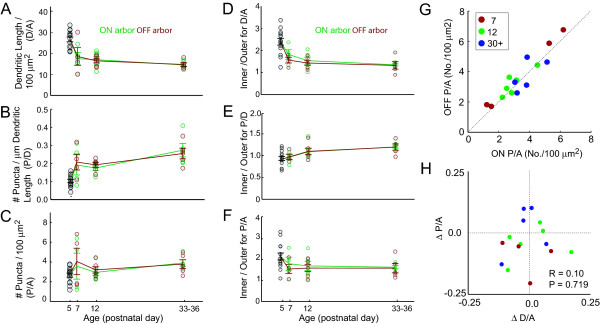
Comparison of ON and OFF arbors of bistratified cells across development. **(a-c) **Global dendritic and puncta densities for bistratified and P5 RGCs. ON arbors are shown in green. OFF arbors are shown in red. Error bars = standard error of mean. **(d-f) **Ratio of dendritic and puncta densities of the inner half of the dendritic arbor divided by the outer half. **(g) **Scatter plot comparing areal density of puncta between ON and OFF arbors of individual cells for all ages recorded. **(h) **Scatter plots showing the relationship of differences in P/A and D/A between the ON and OFF arbors. ΔP/A = [P/A_ON _- P/A_OFF_]/[P/A_ON _+ P/A_OFF_]. Similar calculations were obtained for D/A (ΔD/A). R = correlation of coefficient. *P*-values indicate the probability of producing the given R-value from a random pairing of the data.

Our analysis thus far has focused on the synaptic distributions of monostratified cells across development, which is likely to include more than one type of RGC. Bistratified cells, however, provide an opportunity for us to compare the dendritic and synaptic fields of two dendritic arbors within the same cell. To separate the ON and OFF arbors of a bistratified cell, each cell was digitally rotated so that its arbors were orthogonal to the z dimension. We then plotted the distribution of dendritic length along the z dimension and identified the division between ON and OFF arbors by the trough between the two peaks. Dendritic density, areal synaptic density and linear synaptic density are plotted for the ON and OFF arbors of each bistratified cell at various ages in Figure 8. Overall, both the ON and OFF arbors of bistratified cells appeared to follow the same developmental patterns as monostratified cells, although the changes in D/A and P/D were not as pronounced (Figure 8a–f). Bistratified cells, however, exhibited a shallower centro-peripheral gradient compared to monostratified cells: from P7 onwards, the gradient in areal puncta density was significantly lower in the arbors of bistratified cells (1.66 ± 0.10), compared to monostratified cells (1.91 ± 0.08; *P *= 0.015).

We then compared the areal density of puncta of the ON and OFF arbors of individual bistratified cells. Cells with relatively high P/As in one arbor tended to have similarly high P/As in the other arbor (Figure 8g). In order to determine whether the density of P/A is determined solely by dendritic density, we next tested whether the arbor of a given bistratified cell with the higher dendritic density necessarily had the higher areal synaptic density. Differences in dendritic density (ΔD/A), areal puncta density (ΔP/A) and linear puncta density (ΔP/D) between the ON and OFF arbors of bistratified cells were quantified by subtracting measures for the OFF arbor from those of the ON arbor and dividing the result by the sum of the two arbors. The relationship between ΔD/A and ΔP/A is shown in Figure 8h. If dendritic density drives areal puncta density, then we would expect a positive correlation between ΔD/A and ΔP/A. However, there was no correlation between ΔD/A and ΔP/A (correlation coefficient; R = 0.10, *P *= 0.719; Figure 8h). The lack of correlation between puncta and dendritic density of the two arbors of bistratified cells is consistent with our earlier observation that areal puncta density is not altered by developmental changes in dendritic density.

## Discussion

### Synaptic identity of PSD95-YFP puncta

Our study relies on visualizing the distribution of glutamatergic synapses onto RGCs by expression of fluorescently tagged PSD95, a scaffolding protein found at these excitatory synapses. There are three major issues that need to be addressed when using fluorescent fusion proteins to label synapses: are YFP puncta synaptic? Are all synapses labeled? Does expression of the protein alter synapse number? We have attempted to address all these issues in control experiments that together provided confidence that the expression patterns we observed indicate that PSD95-YFP is an acceptable marker of glutamatergic postsynaptic sites in RGCs.

Studies in a number of systems have used fluorescently tagged PSD95 to label glutamatergic synapses [13-16,25,26]. We tested whether PSD95-YFP could be used to label synapses in mouse RGCs by measuring its colocalization with the presynaptic ribbon marker anti-CtBP2 and found significantly more colocalization in the transfected distribution of PSD95-YFP puncta than in a random redistribution of the same puncta. While the density of synapses in the retinal neuropil makes it difficult to extrapolate from our immunolabeling results an exact number of PSD95-YFP puncta participating in synapses with bipolar cells, studies in culture in which synapse density is much lower have found 87.6% of PSD95-YFP puncta to be colocalized with synaptophysin [27]. We also cannot conclude from our results that all identified puncta at early ages represent synapses. However, it is unlikely that the pattern of puncta observed at P5 and P7, which resembles that observed in the adult, would be produced by the random aggregation of PSD95-YFP.

We noticed that puncta near the center of the cell's dendritic arbor were, on average, brighter than puncta within the peripheral arbor. This decrease in brightness in the distal dendrites may be due to the time required for PSD95-YFP transport. We took this dimming into account in designing the dot-finder program (see Materials and methods). Nevertheless, such a difference in brightness does raise some concern that peripheral puncta were incompletely labeled and, therefore, may have artificially produced centro-peripheral gradients in synapse density. However, the presence of punctate YFP throughout the dendritic arbor of transfected RGCs suggests that protein was available for clustering in proximal and distal dendrites. Furthermore, the linear density of puncta increased with age as arbors became larger rather than decreased as would be expected if the density of puncta were being constrained by transport rates. When our results are compared to those of other studies, the number of puncta per micrometer of dendritic length we measured was within the range of synaptic densities reported for other mammalian RGCs [5,10,11,28-34]. Thus, it is unlikely that we have missed large numbers of endogenous PSD95 sites in the peripheral arbor.

Finally, we took care to avoid expressing PSD95-YFP at levels that potentially could disrupt RGC anatomy and physiology. Low levels of expression appear not to affect synapse size or composition [27]. However, sufficient overexpression of PSD95 can cause increases in EPSC amplitudes, spine diameters, and possibly spine number in hippocampal cultures [25,35]. We therefore reduced the ratio of PSD95-YFP to td-Tomato used in the coating of bullets, limited expression time to 24 hours, and avoided imaging cells with high cytosolic levels of PSD95-YFP. The results of our physiological comparisons of transfected and untransfected cells suggest we obtained an expression level that did not produce the kind of physiological changes that are likely to accompany changes in synapse size or number. Furthermore, the morphologies of our transfected cells were consistent with those reported for comparable ages in previous studies [19,36].

### PSD95-YFP clustering and the emergence of functional inputs

BC ribbon synapses in the IPL are not observed until P11 in mice [37]. However, sEPSCs have been detected in RGCs as early as P7 [18], suggesting that functional synapses form between BCs and RGCs prior to the appearance of ribbons. We also recorded sEPSCs at P7, but observed no sEPSCs at P5 even though, at this age, PSD-95-YFP puncta were apparent and distributed across the RGC dendritic arbor. Our observation of an early synaptic marker such as PSD95 forming puncta at P5 with a delay of two days before sESPCs occur in RGCs is consistent with some previous work. While live imaging studies in hippocampal cultures have revealed that functional synapses can be assembled within an hour of initial contact [27,38], in developing tissue there is often a delay of several days between the first signs of synaptic differentiation and the emergence of fully functional synapses [3]. This delay may reflect the incomplete maturation of the presynaptic release machinery. Indeed, immunolabeling for vesicular glutamate transporter 1 in the retina is observed in only a few BCs at P5 and progressively becomes more apparent, filling in the OFF sublamina by P8 and subsequently the ON sublamina by P10 [39]. In addition, in electron microscopy studies of the developing cat retina, membrane specializations have been observed at cell appositions prior to the presence of synaptic vesicles [40]. Furthermore, in hippocampal cultures at seven days *in vitro*, unlike older cultures, PSD95-YFP clusters have been observed initially unapposed to presynaptic labels but which later appear to induce presynaptic vesicle clustering [41]. The delay between the appearance of PSD95-YFP puncta and detectable sEPSCs might also be due to insufficient clustering of glutamate receptors at synaptic sites. PSD95 has been observed to cluster at postsynaptic sites before the clustering of NMDA and AMPA receptors in cultured hippocampal cells [42]. However, prior to the emergence of sEPSCs, we found that RGCs could be depolarized by exogenous application of the glutamate receptor agonist kainic acid, suggesting that ionotropic glutamate receptors were present in the plasma membrane of RGCs. Finally, it remains possible that at P5, NMDA receptors were clustered at synapses in the absence of AMPA receptors [43].

### Glutamatergic synapses and RGC dendritic stratification

During the first postnatal week, the dendritic arbor of mouse RGCs initially diffusely occupies the depth of the IPL, but over time becomes tightly stratified and restricted to specific sublaminae [19]. This process of stratification has long been suggested to be regulated by glutamatergic transmission; *in vivo *application of the mGluR6 receptor agonist APB prevented cat RGC arbors from undergoing stratification [44,45]. However, our current and previous observations in the mouse retina suggest that this mechanism is unlikely to underlie the initial stages of RGC dendritic stratification in the mouse. First, the axons of presynaptic BCs begin to branch in the IPL at P3 but do not demonstrate significant terminal arbors until about P5 [46], at which time RGC dendritic arbors have undergone significant, albeit not yet complete, lamination [19]. Second, although we found that PSD95-YFP clusters are present and distributed across the RGC arbors by P5, robust glutamate mediated synaptic transmission onto RGCs begins only around P7, when dendritic arbors are already clearly stratified. Furthermore, even before P7, the small difference in puncta and dendrite stratification was not significantly greater than that observed in the adult. It remains possible, however, that glutamatergic inputs are important in further refining and maintaining stratification after the initial lamination process has occurred [47].

### Mechanisms regulating synaptic distributions of RGCs

During the developmental period when RGCs receive functional inputs from BCs, dendritic arbors both grow in size and reduce their density of branching. This decrease in dendritic density with age is greater near the center of the arbor, which results in a flattening of the dendritic density profile (Figure 5b). Similar patterns of exuberant dendritic branching have been reported for RGCs during synaptogenesis in other species [48-50], and likely reflect a transition of the dendritic arbor from an exploratory state to the mature state. Despite these dendritic changes with maturation, the global areal density of postsynaptic densities remained constant. This dissociation was also evident when we compared the distribution of dendrites and synapses onto the ON and OFF arbors of bistratified cells. The lack of a consistent relationship between dendritic density and synaptic density has been implied in past studies of adult RGCs where local peaks in receptive-field sensitivity [51] and synaptic density [12] fail to align with peaks in dendritic density. Thus, there appears to be a clear limit in the extent to which dendritic morphology can be used to make predictions about the density of contacts that are made, and thus synaptic integration, by the cell.

It cannot be assumed from the developmental stability in areal density that all synaptic contacts formed are maintained. Our analysis indicated that there is not a decrease in the population of dendritic segments without puncta with age. This implies that the developmental pruning of dendritic density involves the removal of dendritic segments that had previously formed contacts. Time-lapse imaging studies of labeled RGCs and their inputs will be required to test this possibility.

How, then, might the areal density of inputs be maintained while the dendritic arbor and synapses remodel? When dendritic surface area is the primary limiting factor in the number of synapses formed, an increase in dendritic density would be expected to result in an increase in synaptic density. If, however, there is a limited number of potential inputs that BCs can make and the RGC dendritic density at a given age is more than sufficient to contact all of these BC terminals, then further increases in dendritic density would not be expected to increase synapse number. Studies of the adult retina suggest that BCs could provide such a presynaptic limit to synaptic patterning. Within a given subtype, BC axon terminals tile with limited overlap [5,7,8,52] (Additional file 1) and each axon forms a relatively constant number of synapses [22,24,53]. However, if BC terminals were the limiting factor in the areal density of connections between BCs and RGCs, then the density of their potential inputs would need to be established during the first postnatal week of development.

A centro-peripheral gradient in synaptic number per area also emerges early in development and is maintained despite changes in the dendritic structure and ongoing synaptogenesis. Synaptic gradients are observed to varying extents in the adult RGCs of many species [5,10,54-56] and are thought to be, in part, responsible for the dome shaped receptive field of large field RGCs [12]. Comparisons of synapse number to dendritic surface area in ON alpha RGCs have found that the synaptic gradient is matched to a gradient in the distribution of dendritic surface area [32]. However, the dissociation we observed between synaptic and dendritic gradients across development suggests that there may be additional mechanisms underlying these gradients during synaptogenesis. One possibility is that competition between dendritic arbors of overlapping RGC arbors of the same subtype may result in a reduced number of contacts each RGC neighbor receives from a given BC. The large monostratified alpha cells in the cat form mosaics in which the borders, but not the centers, of their dendritic territories overlap [57] (Additional file 6a). In such cells, the periphery, but not the center, of the cell's dendritic field would be competing with RGCs of the same subtype for BC inputs (Additional file 6b). For a given BC whose axonal terminal lies within the region of dendritic overlap between neighboring RGCs, a smaller percentage of the bipolar cell inputs will be devoted to each of the RGCs. A consistent overlap in RGC tiling across development could then be the source of the stable gradient in puncta areal density observed here. While the change in overlap of RGC dendritic mosaics is not known for mice, the overlap of large-field monostratified RGCs is constant throughout glutamatergic synaptogenesis in the ferret [58]. In contrast to monostratified cells, the dendritic territories of bistratified direction-selective cells in the rabbit retina have little overlap [59]. This difference in tiling is consistent with the relatively lower synaptic gradient of bistratified cells compared to monostratified RGCs observed here. Taken together, we suggest a common mechanism, dendritic competition for a limited field of presynaptic inputs, for establishing the global and local patterns of glutamatergic inputs onto RGCs.

## Conclusion

In summary, adult-like patterns of glutamatergic postsynaptic densities in RGCs emerge early in synaptogenesis, before functional maturation of BC ribbon synapses and before the structural maturation of the RGC dendritic arbor. These spatial synaptic patterns may be shaped by an early mosaic organization of the dendritic fields of RGCs and axonal fields of BCs, rather than derived from a protracted period of dendritic and synaptic remodeling.

## Materials and methods

All experiments were carried out under University of Washington's Animal Care and Use Committee (IACUC) guidelines.

### Cell transfection

C57BL/6 mice were anesthetized with 5% isofluorane and rapidly decapitated. Eyes were removed and placed in cold mouse artificial cerebral spinal fluid (mACSF (in mM): NaCl (119), KCl (2.5), MgCl_2 _(1.3), CaCl_2 _(2.5), NaHPO_4 _(1), glucose (11), HEPES (20), pH 7.37). Retinas were removed from the eyecup and mounted RGC side up on nitrocellulose filter paper (Millipore, Bedford, MA, USA). A Helios gene gun (Bio-Rad, Hercules, CA, USA) was used to ballistically deliver plasmid-coated gold particles. DNA-coated gold particles were prepared by coating [60] 12.5 mg of 1.0–1.6 μm gold particles (Bio-Rad) with approximately 20 μg CMV: td-Tomato and approximately 7 μg CMV: PSD95-YFP [61] plasmids. The tissue was then placed in an oxygenated incubator and maintained at room temperature for several hours before being heated to 33°C. Once expression levels were judged sufficient for imaging (18–24 hours post-transfection), the retinas were fixed for 30 minutes in 4% paraformaldahyde in mACSF, pH 7.4. The data in this study were obtained from 9 P5 retinas, 12 P7 retinas, 9 P12 retinas and 12 P33–35 retinas.

### Immunolabeling

For immunolabeling, tissue was embedded in 4% low melting point agarose after fixation and cut into 60 μm thick sections using a vibratome. Sections were incubated in 1:1,000 mouse anti-CtBP2 (BD Transduction, Franklin Lakes, NJ USA) in 5% normal goat serum and 0.5% Triton-X overnight. The sections were then washed and incubated with an anti-mouse Alexa 633 secondary antibody (Molecular Probes, Carlsbad, California, USA) for two hours.

### Imaging

Images were acquired with Olympus FV scanning confocal microscopes using a 1.4 NA 60× oil objective. Images were acquired at 0.103 × 0.103 × 0.3 μm (FV-500, FV-1000) or 0.115 × 0.115 × 0.3 μm (FV-300) voxel sizes. Although previous studies of mouse RGC maturation did not find significant differences in RGC development with retinal eccentricity [36], the extreme center and periphery of the retina were avoided in order to reduce the effects of centro-peripheral gradients in the maturation of their presynaptic cells, the BCs [46]. The borders of the IPL were identified by imaging reflected laser light and autofluorescence (not shown).

### Image analysis

**Figure 9:**
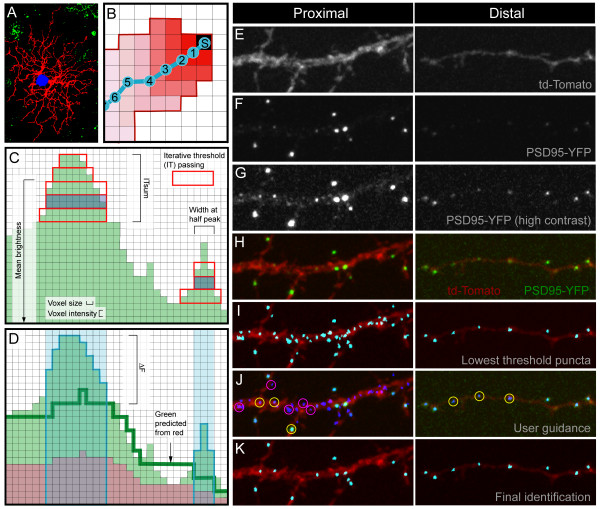
Semi-automated skeletonization and puncta-finding. We illustrate here how we obtained skeletons for the dendrites (a, b), and determined the location of putative postsynaptic sites (c-k). **(a) **A set of masks is generated in Amira to differentiate dendritic regions (masked in red) from the cell body (blue) and background artifacts (green). **(b) **In order to map dendritic length, a dendritic skeleton was generated by first selecting a seed position (S) at an edge of the dendritic mask. This seed initiated a wave of activation that propagated through the dendritic mask, voxel by voxel, until the mask was filled (red shading). The mean positions of this wave front (1–6) were connected to generate the dendritic skeleton (blue). **(c) **Punctate regions of the YFP channel (green) were first identified by thresholding 8 bit images at every other gray value. For each threshold, the red rectangular box represents connected voxels that pass the intensity and size criteria (see Materials and methods). The volume of each punctum was defined by the full-width-half-maximum (blue shading) of the summed rectangles. **(d) **For each potential punctum identified in (c) (shaded blue), the brightness of the YFP channel was compared to the td-Tomato channel to obtain ΔF/F. Red and green values for the non-punctate region of the image were compared to determine a green value predicted by each red value (dark green line). ΔF for each punctum was then defined as the difference between the actual and predicted green value at that punctum, and was divided by the predicted green value to obtain ΔF/F. **(e-k) **Examples of proximal and distal dendrites passing through the user guided stage of puncta identification. **(e) **Dendritic fill with td-Tomato. **(f) **PSD95-YFP clustering. **(g) **PSD95-YFP displayed at high contrast to show dimmer puncta. **(h) **td-Tomato and PSD95-YFP. **(i) **Potential puncta identified with minimal thresholds applied. **(j) **User selection of potential puncta (yellow circles) and artifacts (magenta circles), which are then used to determine a template of optimal thresholds for defining puncta across the entire cell. **(k) **Final output of the dot finder program once optimized thresholds are applied.

Images were processed using Metamorph (Molecular Devices, Sunnyvale, CA, USA), Amira (Mercury Computer Systems Inc. Chelmsford, MA USA), and Matlab (MathWorks, Natick, MA USA). Images were median filtered to reduce photomultiplier tube noise. The contrast and gamma of the images were adjusted to make dim features visible in the figures presented here. Custom Matlab programs were used to generate dendritic skeletons and to identify puncta (Figures 1, 2 and 9, and Additional file 7).

### Skeletonization of dendrites

Dendrites were first identified manually, primarily by thresholding a median filtered image in Matlab or Amira. Cell bodies and debris were digitally highlighted in Amira and removed from further analysis (Figure 9a). Skeletons were then generated from binary masks of the dendrites by drawing segments between adjacent voxels within the mask (Additional file 7). Because thresholding created breaks in fine processes, the mask was first divided into groups of connected voxels. For each group of voxels, the voxel furthest from the group's center of mass was chosen as a seed voxel (Figure 9b). This seed voxel constituted the first point of the first line segment of the dendritic skeleton. The second point on this line segment was obtained by 'activating' all immediately adjacent voxels within the mask and then finding their mean position. These activated voxels activated their adjacent voxels in turn, thereby propagating a wave throughout the mask. With each step of activation, the mean position of the wave was added to the skeleton until the entire mask had been activated. The skeleton was then smoothed by reducing the number of nodes until each segment was approximately 0.5 μm. Small breaks in the skeleton (<3 μm) resulting from breaks in the mask were automatically bridged by additional segments.

### Defining dendritic territories

The dendritic territory of a RGC is the two-dimensional area of retina covered by its dendrites. For our study, each dendritic territory was defined by first mapping its skeleton onto a two-dimensional matrix of 1 μm × 1 μm pixels. This map was then dilated by convolving it with a 10 μm diameter disk and binarizing the result. Gaps in the dilated territory were then filled by performing a morphological closing operation (dilation then erosion) using the same 10 μm diameter disk. Finally, any group of pixels completely surrounded by the product of the closing operation was included in the territory. Examples of territories resulting from this operation can be seen in the Results section.

### Puncta identification

PSD95-YFP puncta were identified in three dimensions using a semi-automated custom Matlab program. Several measures were first collected automatically for all potential puncta from a given cell. We then manually sorted a subset of these potential puncta into positives (potential postsynaptic densities) and negatives (artifacts). The combination of thresholds for three criteria that optimally separated the positives from the negatives was then applied to all potential puncta of that cell. Thousands of puncta could thereby be identified and measured in three dimensions with relatively little user input.

For the automated phase of dot finding, images were first median filtered to reduce noise. The YFP channel was then thresholded at every other gray value (255 to 0) and each thresholded image searched for objects consisting of between 3 and 343 voxels (size criteria; see 'Imaging' for voxel size; Figure 9c). The binary results of each thresholding were then summed to create a three-dimensional map of each potential punctum, whose values corresponded to the number of times the potential puncta passed threshold. The full-width-half-maxima of these sums were defined as the punctum's volume. Adjacent puncta that merged at lower thresholds were identified as separate puncta according to the position of their peaks. A measure of punctum contrast was obtained by dividing the number of times each punctum passed threshold by the mean brightness of that punctum.

The td-Tomato signal was then used to make a prediction of the expected brightness of cytosolic YFP for a given region of the dendrite to which the actual brightness of a punctum could be compared (Figure 9d). Within non-punctate regions of the dendrite, each brightness value of voxels in the red channel corresponds to some value in the green channel; this relationship enables us to predict the brightness value for the green channel within the punctum based on the brightness value of the red channel at the punctum. The ΔF for the puncta was then defined as the mean difference from the predicted values of the brightest half of the puncta's voxels. The ΔF was then divided by the predicted brightness to obtain ΔF/F.

For the user-guided stage of puncta identification, a maximum intensity projection of the cell with all potential puncta labeled was generated from which the user could select a set of example positives and negatives (Figure 9e–j). These positives and negatives were chosen so as to include relatively dim puncta in the periphery of the arbor and to exclude irregularly shaped artifacts present in the cytosol of larger processes. Because some cells exhibited a centro-peripheral gradient in puncta brightness, a second measure of ΔF/F was generated that was scaled relative to a linear model of the relationship of ΔF/F and distance to the cell body (Figure 9e–h). A set of thresholds for contrast, ΔF/F and scaled ΔF/F were generated by finding the combination of thresholds that separated user identified positives from negatives with the smallest number of errors. This set of thresholds was then applied to all puncta. Additional iterations of user guidance could be used to improve the sorting of puncta; however, one or two rounds of user guidance were generally sufficient to sort puncta (Figure 9k).

### Colocalization

Colocalization of PSD95-YFP puncta with the presynaptic protein CtBP2 was quantified by comparing the colocalization of the observed distribution of PSD95-YFP puncta to that of a randomized distribution. We defined an area around each potential postsynaptic site in which to search for presynaptic signal by dilating the volume of each identified PSD95-YFP puncta by 0.3 μm in three dimensions. Presynaptic sites were then defined by thresholding each optical plane in the CtBP2 channel to include all voxels that were at least 10% brighter than the median brightness of all voxels at that depth (all planes within ± 0.6 μm of z depth). Monte Carlo simulation was then used to determine what the rate of colocalization between PSD95-YFP puncta and CtBP2 signal would be if the PSD95-YFP puncta were randomly distributed. For each cell, dilated PSD95-YFP puncta were randomly rearranged across the surface of the cell's dendritic arbor 10,000 times. The number of overlapping voxels required to count a PSD95 puncta as colocalized with the CtBP2 signal was determined by finding the minimum number of overlapping PSD-95-YFP and CtBP2 voxels that would result in less than 50% of a cell's randomized puncta being counted as colocalized.

### Statistics

Except where otherwise noted, we used Wilcoxon rank sum tests to assess statistical differences between groups. One-way ANOVA was used to compare values across ages. Paired tests were performed using a paired-sample sign test. R-values are correlation coefficients produced by dividing the covariance between two measures by the square root of the product of the covariance within each measure. The *P*-value associated with each R-value indicates the probability of observing an equal or greater R-value from a random pairing of measures (as determined by bootstrap analysis).

### Electrophysiology

#### Tissue preparation and RGC identification

Retinal slice preparation was performed as previously described [62]. RGC somata were identified based on their location in the ganglion cell layer and their large diameter. The fluorescent dye, sulforhodamine B (0.005%; Sigma, St. Louis, MO, USA), was included in the intracellular solution. For whole-mount preparations, retinas were transfected with td-Tomato and PSD95-YFP as described above. After incubation, retinas were removed from filter paper and mounted on a plastic slide ganglion-cell-side-up in 1% low melting point agarose. Both td-Tomato-fluorescent and non-fluorescent RGC bodies were targeted. During the recording, RGCs were filled with Lucifer Yellow (0.05%; Sigma). We recorded only from cells with both an obvious dendritic arbor and axon.

#### Whole cell recordings

Whole-cell recordings were made from RGCs in both the retinal slice and in the whole mount preparation. Before a RGC in the whole mount preparation was accessible for the patch electrode, the fiber layer was removed above the cell using another electrode. The resistance of the patch electrodes with standard solutions usually ranged from 4–6 MΩ. Liquid junction potentials of 15 mV were corrected before the cell was attached. Series resistance and capacitance of pipettes as well as cell capacitance were not compensated. Seal resistances of 1 GΩ and higher were routinely obtained. sEPSCs were recorded from RGCs voltage-clamped at -60 mV, the reversal potential for currents through chloride-permeable channels. Spontaneous inhibitory postsynaptic currents were recorded at 0 mV, the reversal potential for currents through non-selective cation channels. Postsynaptic currents were recorded for a two minute period. The holding current was frequently monitored during an experiment, and recordings with shifting holding currents were aborted. All experiments were carried out at room temperature (20–22°C).

In some experiments, the glutamate receptor agonist kainic acid (10 μM, Sigma) was puffed onto the slice preparation using a Picospritzer System (General Valve Corporation, Fairfield, NJ, USA). The puffer pipette (filled with extracellular solution) was positioned so that the inner plexiform layer in proximity to the patch-clamped cell body was superfused. In this set of experiments, cadmium chloride (250 μM; Sigma) was added to the extracellular solution to block synaptic activity.

Data acquisition was performed with an Axopatch 200B amplifier with a frequency of 5 kHz using the Clampex software (Axon Instruments, Foster City, CA, USA). Signals were 2 kHz Bessel filtered.

#### Data analysis

sEPSCs were analyzed using Mini Analysis (Synaptosoft, Dectaur, GA, USA). All events were selected so that the rise and decay phases did not overlap. The median amplitude and mean frequency were obtained for each cell. In addition, we used the Clampfit 8.1 software (Axon Instruments) to measure the amplitude of kainic acid-evoked currents. Subsequent data processing was performed with the Origin 6.0 software package (MicroCal, North Hampton, MA, USA).

#### Solutions

Retinas were stored in physiological extracellular saline solution containing (in mM): NaCl (125), KCl (2.5), MgCl_2 _(1), NaH_2_PO_4 _(1.25), glucose (20), NaHCO_3 _(26) and CaCl_2 _(2). To maintain a physiological pH of 7.4, the solution was continuously bubbled with 95% O_2 _and 5% CO_2_. The intracellular patch clamp solution contained (in mM): cesium gluconate (120), CaCl_2 _(1), MgCl_2 _(1), Na-HEPES (10), EGTA (11), and TEA-Cl (10), adjusted to pH 7.2 with CsOH. The calculated chloride equilibrium potential (E_cl_) was -59.3 mV whereas the equilibrium potential for currents through non-selective cation channel was 0 mV.

Neurotransmitter receptor antagonists CNQX (20 μM), AP-7 (40 μM), SR-95531 (5 μM), TPMPA (75 μM), and strychnine hydrochloride (5 μM) were dissolved in extracellular solution and bath-applied to the recording chamber by a gravity superfusion system. All chemicals were obtained from Sigma.

## Competing interests

The author(s) declare that they have no competing interests.

## Authors' contributions

JLM performed the imaging and image analysis in this study. TS performed the electrophysiological recordings and their analysis. ROLW participated in the design and coordination of the work. All authors contributed to the preparation of the manuscript and approved the final manuscript.

## Supplementary Material

Additional file 1 Connectivity with bipolar cells shapes excitatory center of RGC receptive fields. **(a) **The lateral extent of the RGC dendritic arbor and its connectivity with bipolar cell axon terminals largely determines the region of visual space sampled by the RGC. Blue rectangle indicates the lateral extent of the receptive field center for RGC in white (G). P, cone photoreceptors; B, bipolar cells. IPL, inner plexiform layer. **(b) **The number of synapses (red dots) per unit area of retinal surface between a RGC and the mosaic of bipolar cell axons (shaded polygons) varies across the dendritic field of large-field RGCs. Blue outline denotes the extent of the receptive field center of this RGC. **(c-f) **Centro-peripheral gradients in the density of connections between a RGC and the mosiac of bipolar cell axon terminals contributes to RGC receptive fields that are more sensitive to a visual stimulus at their centers. (c) Representation of the gaussian-like receptive fields of individual BCs (red and green) in the background of the receptive fields of the population of BCs of the same subtype. Each receptive field reflects the sensitivity of a given BC to the region of visual space represented along the x-axis. (d) The number of BC inputs (y-axis) onto a single RGC at different regions of its dendritic field (x-axis). Synaptic density is highest at the center of the RGC dendritic field. Alternating gray and white bars indicate the axonal territories of adjacent bipolar cells. (e) The relative weighting of bipolar cell receptive fields by a RGC with the connectivity pattern shown in (d). (f) Excitatory receptive field of the RGC calculated by summing the weighted receptive fields in (e). (c-f) Adapted from [12]. Click here for file

Additional file 2 RGC rotation. Rotation of the P7 bistratified RGC shown in Figure 1. Click here for file

Additional file 3 PSD95-YFP puncta on primary dendrites of RGCs. PSD95-YFP puncta are found on primary dendrites of **(a, b) **some, but **(c) **not all OFF RGCs. This is particularly apparent in the orthogonal rotations of the image stack (right panels). Click here for file

Additional file 4 P22 RGC with a large dendritic arbor in the ON sublamina and a small dendritic arbor stratifying within the OFF sublamina. PSD95-YFP puncta are restricted to the two sublaminae within the IPL at which dendrites stratify. Note the absence of PSD95-YFP puncta within the dendritic segment traversing the two sublaminae. Click here for file

Additional file 5 Comparison of ON and OFF monostratified cells across development. **(a-c) **Average dendritic and puncta densities for monostratified RGCs. ON cells are shown in green. OFF cells are shown in red. Error bars = standard error of mean. An asterisk indicates a significant difference between ON and OFF cells. (**d-f) **Ratio of dendritic and puncta densities of the inner half of the dendritic arbor divided by the outer half. Click here for file

Additional file 6 Potential relationship between RGC tiling and centro-peripheral gradient in BC synaptic distribution across the RGC arbor. **(a) **Schematic of the cellular mosaic formed by a single type of RGC in which dendritic territories overlap in the periphery but not the center of each dendritic field. **(b) **Illustration of how lower areal densities of synapses (red dots) in the periphery of the RGC dendritic arbor might be the result of dendritic competition for presynaptic contacts. Blue circles represent the lateral spread of an individual BC axonal arbor. BCs in the periphery of an RGC dendritic arbor are more likely to divide their inputs between dendrites of neighboring RGCs of the same subtype. Asterisk indicates soma. Click here for file

Additional file 7 Two dimensional representation of the skeletonization algorithm used to measure the length of dendrites. The red fields represent dendritic segments. A seed pixel (first blue dot) is chosen at an extreme edge of the object. This pixel initiates a wave of activated pixels (green bar) that spreads throughout the object. The mean positions of the wave at each step are connected to generate a skeleton. The number of nodes in the skeleton is subsequently reduced to reduce noise. Click here for file
