# Supplementary figures and images for: Developmental patterning of glutamatergic synapses onto retinal ganglion cells
Source: Neural Dev. 2008 Mar 26;3:8. doi: 10.1186/1749-8104-3-8 (PMC2311295; doi:10.1186/1749-8104-3-8)

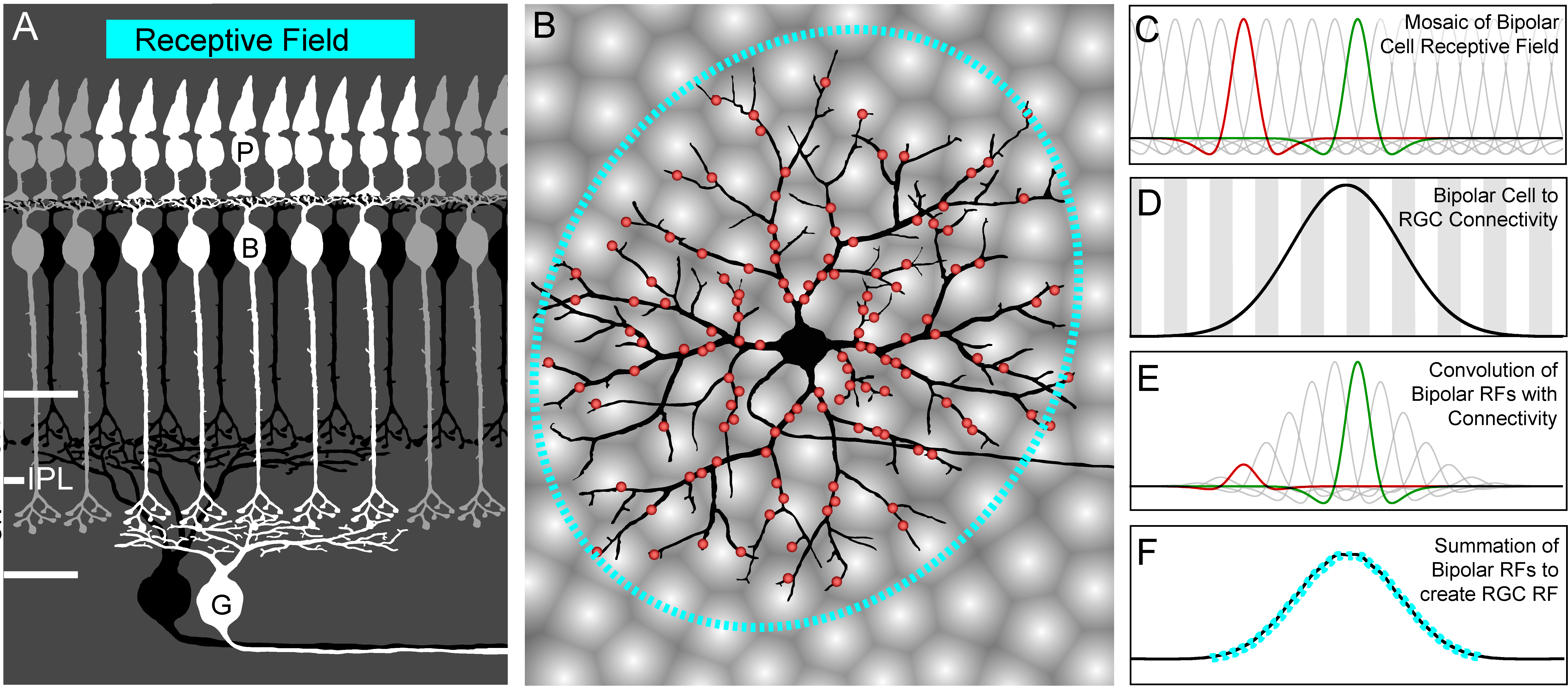

Supplement: Additional file 1 — Connectivity with bipolar cells shapes excitatory center of RGC receptive fields. (a) The lateral extent of the RGC dendritic arbor and its connectivity with bipolar cell axon terminals largely determines the region of visual space sampled by the RGC. Blue rectangle indicates the lateral extent of the receptive field center for RGC in white (G). P, cone photoreceptors; B, bipolar cells. IPL, inner plexiform layer. (b) The number of synapses (red dots) per unit area of retinal surface between a RGC and the mosaic of bipolar cell axons (shaded polygons) varies across the dendritic field of large-field RGCs. Blue outline denotes the extent of the receptive field center of this RGC. (c-f) Centro-peripheral gradients in the density of connections between a RGC and the mosiac of bipolar cell axon terminals contributes to RGC receptive fields that are more sensitive to a visual stimulus at their centers. (c) Representation of the gaussian-like receptive fields of individual BCs (red and green) in the background of the receptive fields of the population of BCs of the same subtype. Each receptive field reflects the sensitivity of a given BC to the region of visual space represented along the x-axis. (d) The number of BC inputs (y-axis) onto a single RGC at different regions of its dendritic field (x-axis). Synaptic density is highest at the center of the RGC dendritic field. Alternating gray and white bars indicate the axonal territories of adjacent bipolar cells. (e) The relative weighting of bipolar cell receptive fields by a RGC with the connectivity pattern shown in (d). (f) Excitatory receptive field of the RGC calculated by summing the weighted receptive fields in (e). (c-f) Adapted from [12]. [file 1749-8104-3-8-S1.tiff]

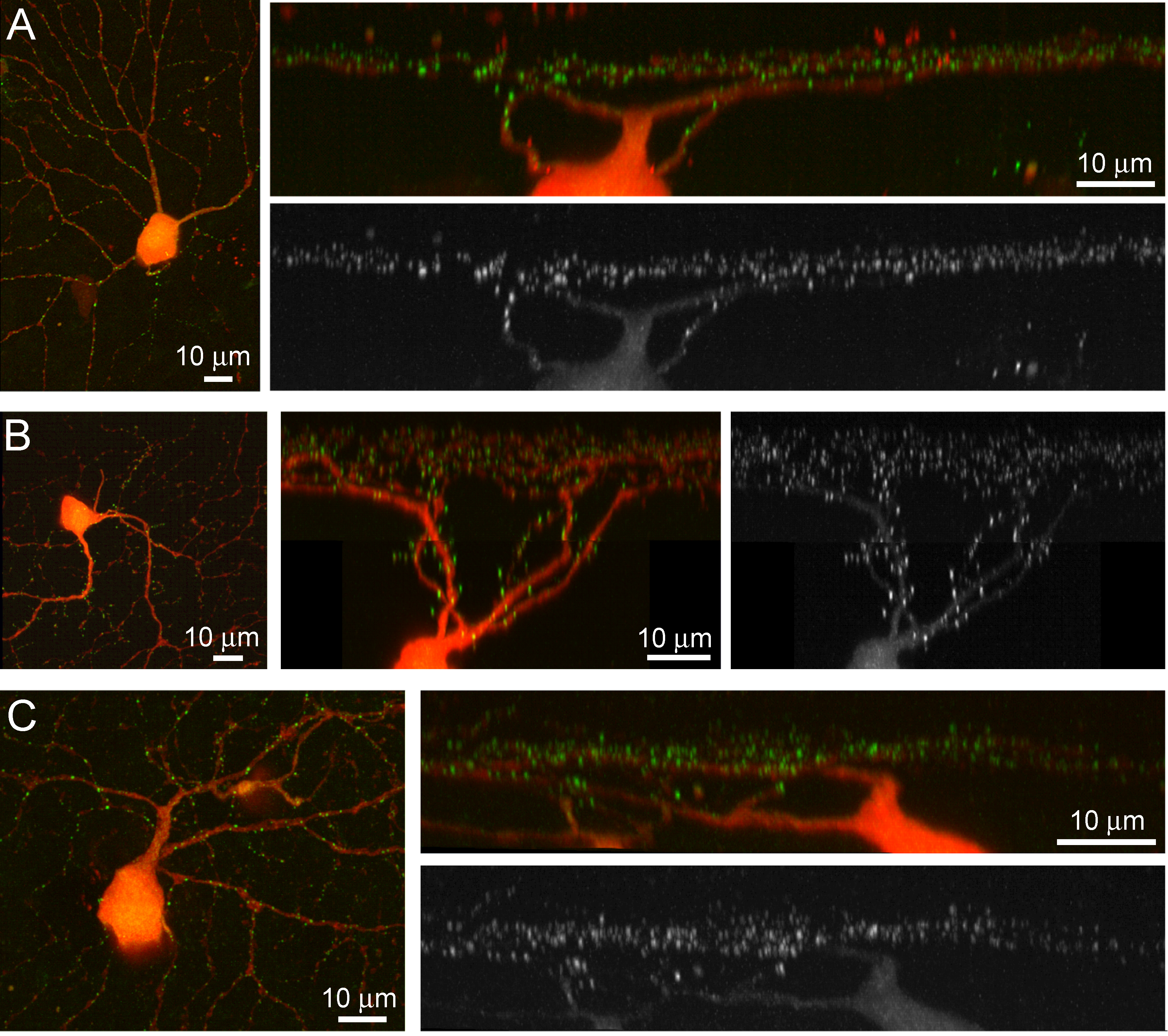

Supplement: Additional file 3 — PSD95-YFP puncta on primary dendrites of RGCs. PSD95-YFP puncta are found on primary dendrites of (a, b) some, but (c) not all OFF RGCs. This is particularly apparent in the orthogonal rotations of the image stack (right panels). [file 1749-8104-3-8-S3.tiff]
